# Photophysical lock-in detection enables background-free upconversion emission imaging

**DOI:** 10.1038/s41377-026-02414-2

**Published:** 2026-09-07

**Authors:** Niusha Bagheri, Chenyi Wang, Du Guo, Anbharasi Lakshmanan, Qi Zhu, Xu Chen, Nahid Ghazyani, Qiuqiang Zhan, Georgios A. Sotiriou, Haichun Liu, Jerker Widengren

**Affiliations:** 1 Department of Applied Physics, Experimental Biomolecular Physics, KTH Royal Institute of Technology, Stockholm, Sweden; 2 Centre for Optical and Electromagnetic Research, South China Academy of Advanced Optoelectronics, South China Normal University, Guangzhou, China; 3 Faculty of Physics, Kharazmi University, Tehran, Iran; 4 Department of Microbiology Tumor and Cell Biology Karolinska Institute, Stockholm, Sweden; 5 Department of Chemistry, Science for Life Laboratory (SciLifeLab), Stockholm University, Stockholm, Sweden

**Keywords:** Imaging and sensing, Nanoparticles

## Abstract

Lanthanide-based upconversion nanoparticles (UCNPs) have attracted considerable attention in biomedical applications, due to their anti-Stokes shifted emission enabling autofluorescence-free signal detection. However, residual excitation light can still interfere with their relatively weak emission signals. While commonly used lock-in detection can distinguish weak signals from substantial random background, concurrently modulated residual excitation light is not eliminated. This remains a challenge, particularly under demanding experimental conditions. Here, we propose a photophysical lock-in detection (PP-LID) approach based on the discovery that UCNPs can act as frequency mixers in response to intensity-modulated excitation. Particularly, excitation modulated at multiple base frequencies can generate additional spectral components at the beat frequencies (BFs) between the base modulation frequencies. These signals are resolvable by frame-rate-limited cameras, devoid of ambient and residual excitation light, and can be adapted through nanoparticle engineering. Extracting BF signals by PP-LID thus provides a strategy to significantly enhance signal-to-background conditions in UCNP-based bioimaging and biosensing.

## Abstract (web summary)

Upon dual-frequency excitation upconversion nanoparticles generate beat-frequency signals owing to their inherent optical nonlinearity. These low-frequency signals are absent in any excitation and background light, enabling background-free camera-based detection.

## Graphical abstract

**Figure:**
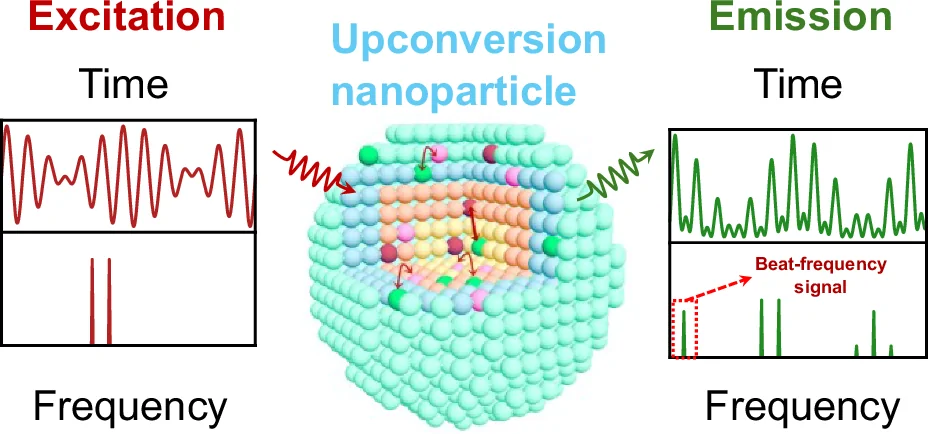

## Introduction

Lanthanide-based upconversion nanoparticles (UCNPs) have attracted significant attention for biomedical imaging and biosensing applications^1,2^. The large anti-Stokes shifts in their emission provide a spectral basis for background-free detection^3^, offering higher sensitivity compared to conventional fluorophores with Stokes-shifted emission. However, the relatively low brightness of UCNPs remains a significant limitation, especially in challenging conditions, such as in small-animal imaging studies with low UCNP concentrations, low excitation power (restricted by ANSI laser safety limits), and large tissue depths. Wide-field deep-tissue imaging imposes optical constraints that reduce the effectiveness of optical filters even of high optical densities (ODs). Such filters are typically designed to achieve their nominal OD value with normal light incidence^4^. In wide-field diffuse optical imaging, emission light reaches the filters over a broad range of incident angles, at which the filter performance can be significantly worse than the nominal OD. This makes suppression of excitation light far more challenging. Therefore, it is meaningful to develop approaches to enhance the signal-to-background ratio (SBR) in UCNP imaging by other, orthogonal principles to fully realize its potential in biological applications.

Lock-in detection (LID) makes it possible to capture weak signals and dramatically increase detection sensitivity by orders of magnitude. LID is widely employed in different optical microscopy and spectroscopy techniques and in applications requiring ultrasensitive optical detection, such as in remote optical sensing^5–7^. LID works by distinguishing the periodic signal generated by the modulated light source, effectively discriminating this signal from random background noise. However, LID does not resolve the issue of residual excitation light, which gets modulated at the same frequency as the optical signal being detected and is thus not suppressed in the detection.

To overcome this drawback, we propose a photophysical lock-in detection (PP-LID) approach, based on excitation modulation and specifically tailored to take advantage of the inherent nonlinearity of upconversion luminescence (UCL). By simulations and experiments we first demonstrate how additional frequency components of UCL signal arise, not present in the residual excitation light background, and how LID at these frequencies can significantly suppress background light and enhance SBR. We then show how excitation modulation at two different frequencies allows LID at their difference frequency, enabling LID with low-frame-rate cameras, and how the relative strength of this beat frequency (BF) signal can be optimized by composition and design of the UCNPs. Finally, we show how the proposed PP-LID approach together with optimized UCNPs enhance SBR in imaging experiments, under high background conditions, and deep into tissues.

## Results

### Second-harmonic signal generation in UCNPs under intensity-modulated excitation

**Fig. 1:**
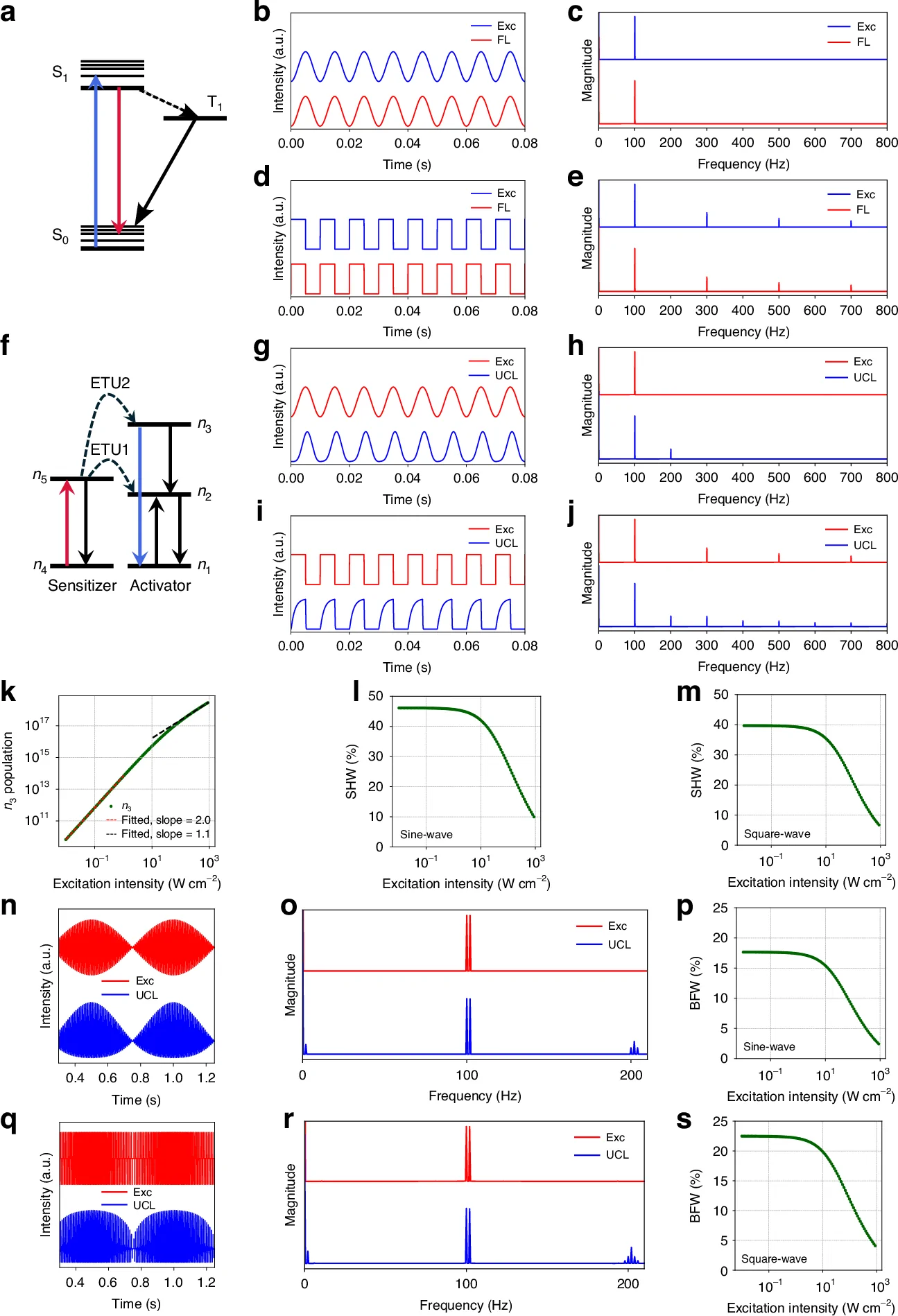
Excitation modulation response of organic fluorophores and upconversion nanoparticles (UCNPs). **a** Jablonski diagram of a typical organic fluorophore, showing light excitation, fluorescence, inter-system crossing, and triplet state relaxation processes. Response of fluorophore fluorescence intensity to modulated excitations: **b** sinusoidal excitation, and **d** square-wave excitation. Square-wave excitation was set with 50% duty cycle in all simulations. Fast Fourier transform (FFT) spectrum of the fluorophore fluorescence signal compared to that of the excitation wave under **c** sinusoidal- and **e** square-wave excitation. The maximum excitation intensity for fluorophore simulations was set at 100 W cm^−2^. **f** Energy level diagram of a representative UCNP, including a sensitizer and an activator yielding a two-step energy-transfer upconversion system. Response of UCL intensity to modulated excitations: **g** sinusoidal excitation, and **i** square-wave excitation. The FFT spectra of the UCL signal compared to that of the excitation wave under **h** sinusoidal and **j** square-wave excitation. **k** Simulated excitation-intensity dependence of the *n*_3_ population (proportional to UCL intensity) under CW excitation. **l** Excitation-intensity dependence of the SHW of the UCL under single sinusoidal-wave excitation. **m** Excitation-intensity dependence of the SHW of the UCL under single square-wave excitation. **n** Response of UCL intensity to dual-sinusoidal-wave modulated excitation. **o** The spectrum of the FFT of the UCL signal compared to that of the excitation wave under dual**-**sinusoidal-wave excitation. **p** Excitation-intensity dependence of the BFW of the UCL under dual sinusoidal-wave excitation. **q** Response of UCL intensity to dual-square-wave modulated excitation. **r** The spectrum of the FFT of the UCL signal compared to that of the excitation signal under dual**-**square-wave excitation. **s** Excitation-intensity dependence of the BFW of the UCL under dual square-wave excitation. The maximum excitation intensity for UCNP simulations was set at 1 W cm^−2^

We first explored our rationale underlying PP-LID through simulations (Fig. 1), comparing the emission response of UCNPs upon sinusoidal and square-wave excitations (Fig. 1f–j) with that of an organic fluorophore with Stokes-shifted emission (Fig. 1a–e). When the fluorophore (Fig. 1a) is exposed to modulated excitation (maximally 100 W cm^−2^ sinusoidal- or square-wave) at relatively low frequencies (<200 Hz), it maintains a linear response to the excitation light and a negligible buildup of dark triplet states^8–12^. The resulting fluorescence signal then follows the excitation pattern, maintaining a sinusoidal (Fig. 1b) or square waveform (Fig. 1d) with frequency components identical to the excitation light (Fig. 1c, e, numerical simulations detailed in Methods).

Unlike organic fluorophores, UCNPs exhibit an inherent nonlinear response to the excitation intensity, also under non-saturation excitation conditions (with excitation intensities, ${I}_{{\rm{exc}}}$, below 1 W cm^−2^), fundamentally rooted in their sequential multi-step excitation process preceding their emission^13,14^. We hypothesized that when exposed to a modulated excitation intensity, UCNPs may thus generate emission signals with additional frequency components, beyond those contained in the excitation light. To verify this hypothesis, we conducted similar numerical simulations as for the fluorophore, based on a representative two-photon energy-transfer upconversion system, including a sensitizer and an activator (Fig. 1f) (detailed in Methods). Upon sinusoidal excitation at frequency f, a second harmonic (SH) signal (2f) was observed in the fast Fourier transform (FFT) spectrum of the UCL (Fig. 1g, h). For square-wave excitation at base frequency f with 50% duty cycle (motivated in section S1 in the SI) the FFT spectrum of the UCL also revealed even-order harmonics (Fig. 1i, j).

To further confirm our predictions, we investigated numerically how the strength of the SH signal depends on the nonlinearity of the upconversion system. In the studied ${I}_{{\rm{exc}}}$ range (0.01–1000 W cm^−2^), the UCL intensity exhibited a progressively weaker power-law dependence on ${I}_{{\rm{exc}}}$ (Fig. 1k), indicating increasing upconversion saturation^15^. We simulated how the second-harmonic weight (SHW), defined as

1 $${\rm{SHW}}=\frac{{I}_{2f}}{\left({I}_{f}+{I}_{3f}\right)/2}\times 100 \%$$

where ${I}_{x}$ (for x=f,2f,3f) represents the amplitude of the UCL at frequency x in the FFT spectrum, varies with ${I}_{{\rm{exc}}}$ under sinusoidal- or square-wave (50% duty cycle) excitation. Below 1 W cm^-2^, where no noticeable upconversion saturation is present, as indicated by a slope efficiency of 2.0 (Fig. 1k), the SHW remains relatively stable (Fig. 1l, m). With further increasing ${I}_{{\rm{exc}}}$, the SHW starts to decrease substantially.

**Fig. 2:**
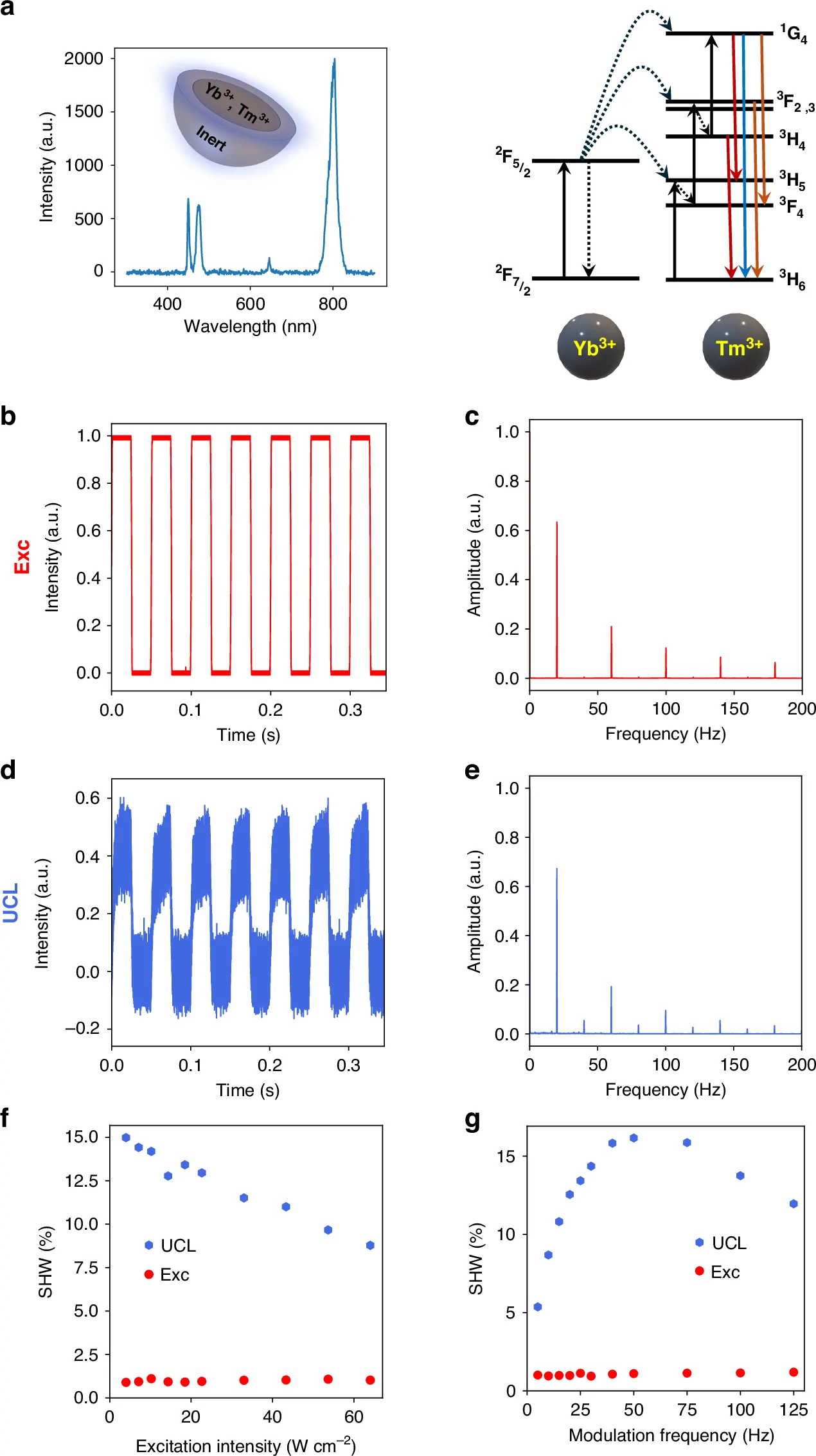
Second-harmonic (SH) signal generation of UCNPs under square-wave excitation. **a** UCL spectrum (Left) and photophysical model (Right) of the studied NaYF_4_:20%Yb^3+^, 0.5%Tm^3+^@NaYF_4_ (**YbTm@Y**) UCNPs under 980 nm excitation. **b** The applied square-wave excitation (50% duty cycle with an excitation intensity within the pulses of 4 W cm^−2^) and **c** its FFT spectrum (**b**). **d** The resulting UCL signal (430–850 nm) and **e** its FFT spectrum. The UCL trace in (**d**) was plotted after smoothing the raw data using a 7-point sliding average. Negative intensity values in the raw UCL trace arises from background subtraction and electronic noise in the detection system rather than negative photon emission. The dependence of the defined SH weight (SHW) (Eq. 1) of the UCL (430–850 nm) on (**f**) the excitation intensity in the pulses and **(g)** the base excitation modulation frequency

To experimentally verify the numerical predictions, we first measured the emission response of NaYF_4_:20%Yb^3+^,0.5%Tm^3+^@NaYF_4_ (**YbTm@Y**) UCNPs (Fig. S1, Fig. 2a)^15^, under square-wave excitation (50% duty cycle), optical setup illustrated in Fig. S3. Figure 2b shows a typical excitation intensity time-trace applied in the experiments (modulated at 20 Hz), with its FFT spectrum including odd harmonics (20, 60, 100 Hz, …) of the base frequency (20 Hz), but negligible even harmonics (Fig. 2c). In stark contrast, in the FFT spectrum of the time trace of the generated UCL (430–850 nm) (Fig. 2d), significant even harmonics (at 40, 80, 120 Hz, …) were observed alongside the original odd harmonics (Fig. 2e), validating our theoretical prediction (Fig. 1j).

Next, we investigated the influence of excitation intensity on the UCL SHW, applying a fixed base modulation frequency, *f* = 40 Hz and varying ${I}_{{\rm{exc}}}$ between 4 and 64 W cm^−2^. As shown in Fig. 2f, the UCL SHW decreases monotonically with increasing ${I}_{{\rm{exc}}}$, qualitatively in line with the trend found in the simulations (Fig. 1m). We also monitored the laser light throughout the experiments and confirmed that the SHW of the UCL is always significantly higher than that of the laser excitation, ruling out the possibility of instrumental artefacts and imperfections in the excitation modulation as a reason for prominent UCL SHWs. Experimental investigations on NaYF_4_:20%Yb^3+^,2%Er^3+^@NaYF_4_ (**YbEr@Y**) UCNPs (Fig. S2) also confirmed generation of even harmonics in the UCL (Fig. S4a–e), displaying a similar dependence on ${I}_{{\rm{exc}}}$ (Fig. S4f) as the **YbTm@Y** UCNPs.

We also investigated the effect of the base modulation frequency. For the **YbTm@Y** UCNPs, the SHW of the UCL increases with higher f, until reaching a maximum, and then decreases (Fig. 2g). The SHW of the UCL of **YbEr@Y** UCNPs displayed a different response to excitation modulation frequency, with a monotonic increasing trend in the tested range (Fig. S4g).

These simulations and experimental results reveal that upconversion systems can generate SH signals upon sinusoidal- or square-wave modulation and that the nonlinearity of the system is a key factor in this process.

### Beat-frequency signal generation in UCNPs under dual-frequency intensity-modulated excitation

The SH signal can thereby offer a unique LID channel for UCNPs, allowing detection of UCL free from background noise and residual excitation light. However, a drawback is its higher frequency value, which according to the Nyquist theorem cannot be resolved by low-speed detectors such as cameras. This drawback prompted us to further explore the ability of UCNPs to generate new, lower frequency components. We realized that the inherent nonlinearity of UCNPs could enable them to function as modulation frequency mixers, generating difference, or BF signals when multiple base modulation frequencies are simultaneously applied to the excitation light. To test this, we extended our simulations to UCNPs excited by two superimposed sinusoidal or square waves, modulated at different (base) frequencies (${f}_{1}$ and ${f}_{2}$). It was found that BF signals were indeed generally generated (Fig. 1n, o, q, r). For ${I}_{{\rm{exc}}}$ < 1 W cm^−2^, in absence of any upconverison saturation, the beat-frequency weight (BFW), defined as

2 $${\rm{BFW}}=\frac{{I}_{{f}_{2}-{f}_{1}}}{\left({I}_{{f}_{1}}+{I}_{{f}_{2}}\right)/2}\times 100 \%$$

where ${I}_{y}$ (for $y={f}_{1},{f}_{2},{f}_{2}-{f}_{1}$) represents the amplitude at frequency y in the FFT spectrum, remains almost constant. Like for the SHW, the BFW then starts to decrease more significantly at further elevated ${I}_{{\rm{exc}}}$ (Fig. 1p, s).

**Fig. 3:**
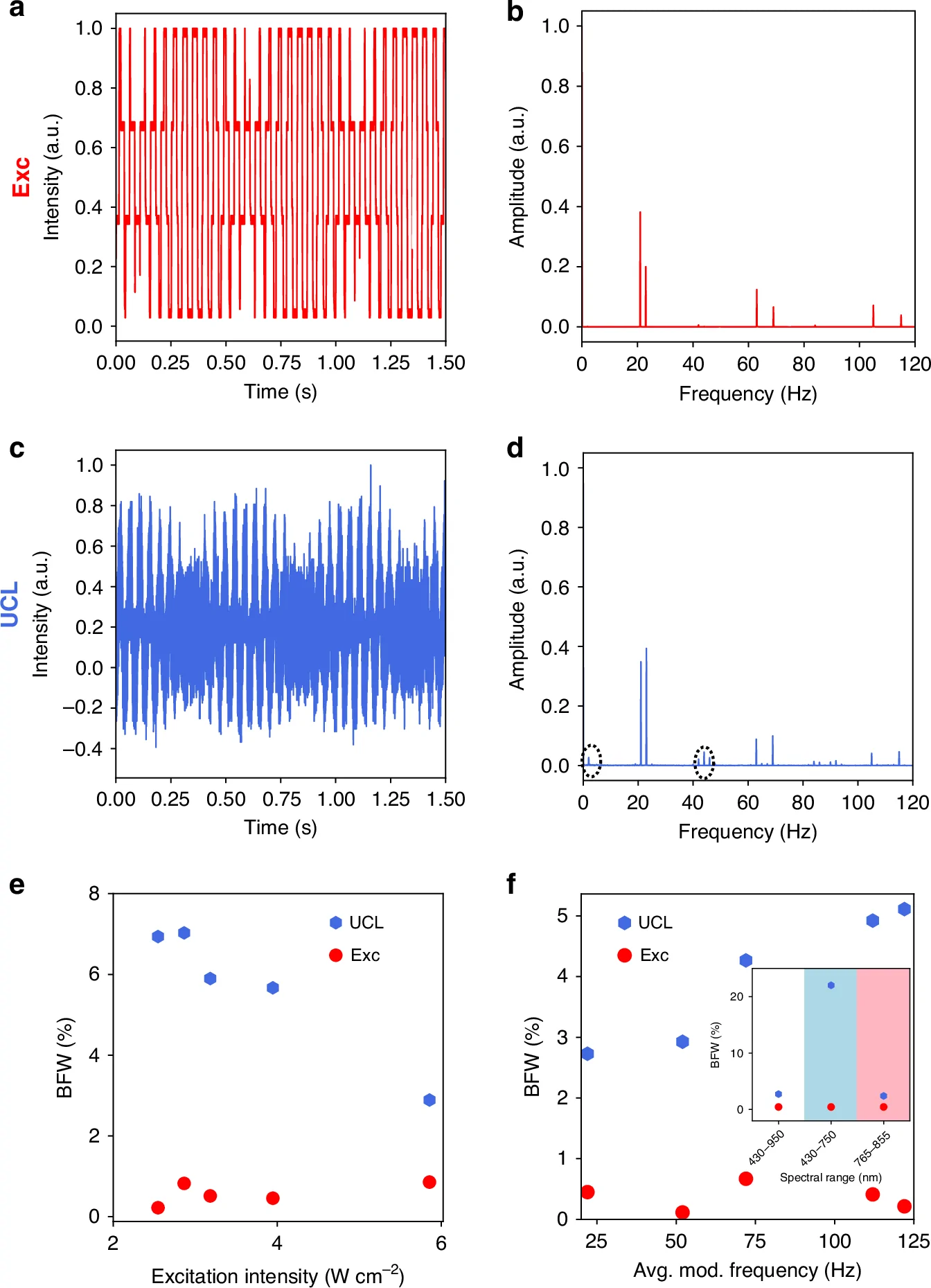
Beat frequency (BF) signal generation of UCNPs under dual square-wave excitation. **a** Recorded excitation intensity trace output from the two merged 980 nm laser beams modulated at 21 and 23 Hz, respectively. **b** FFT spectrum of the excitation intensity trace in (**a**). **c** Intensity trace of the generated UCL signal (430–850 nm) of **YbTm@Y** UCNPs (time averaged excitation intensity: 2 W cm^−2^, each laser), and **d** its FFT spectrum. Negative intensity values in the raw UCL trace arises from background subtraction and electronic noise in the detection system and indicate fluctuations around a zero-referenced baseline rather than negative photon emission. **e** The dependence of the defined beat-frequency weight (BFW) (Eq. 2) of the UCL (430–850 nm) on the time averaged excitation intensity (each laser) in the pulses. **f** The dependence of the BFW of the UCL signal (430–850 nm) of **YbTm@Y** UCNPs on the average modulation frequency ($({f}_{1}+{f}_{2})/2$). Time averaged excitation intensity: 6 W cm^−2^, each laser. Inset: The BFWs of the UCL signal in different spectral ranges (${f}_{1}=21$ Hz and ${f}_{2}=23$ Hz)

Guided by these simulations, we then experimentally studied **YbTm@Y** UCNPs under excitation of two 980-nm laser beams with equal power and modulated at different frequencies, using a modified optical setup (Fig. S5). Figure 3a, c show the recorded excitation and UCL (430–850 nm) intensity waveforms with the laser excitation beams separately modulated at 21 and 23 Hz, respectively. As expected, the FFT spectrum of the (merged) excitation light reveals the two base modulation frequencies along with their corresponding odd harmonics (the third and fifth harmonics shown in Fig. 3b), with negligible other frequency components. However, in the FFT spectrum of the UCL (Fig. 3d) the BF signal (at 2 Hz) together with the sum frequency signal (at 44 Hz) were also clearly detected, in addition to the expected base frequency components along with their respective SH signals (at 42 Hz and 46 Hz).

Next, we investigated ${I}_{{\rm{exc}}}$ effects and found that the BFW of the UCL generally decreases with increasing ${I}_{{\rm{exc}}}$ in the tested range (2–6 W cm^−2^) (Fig. 3e), qualitatively in line with the trend found in the simulations (Fig. 1s). We also separated the two-photonic 800 nm emission band of Tm^3+^ from multi-photonic emissions (430–750 nm) and analyzed their responses to dual-base-frequency modulation (21 and 23 Hz). The much stronger BFWs generated in the multi-photonic UCL bands (>20%), compared to the two-photonic 800 nm emission (*~*3%, inset, Fig. 3f) indicate that the ability of UCNPs to generate BF signals in response to superimposed modulated excitation waves is also correlated with their inherent nonlinearity. When varying the two base modulation frequencies while maintaining a fixed difference frequency of 2 Hz, a BF signal is consistently generated, with a slightly increasing trend (Fig. 3f). Investigations on other UCNPs, such as **YbEr@Y**, further validate the ability of UCNPs in generating BF and sum frequency signals (Fig. S6).

In addition to the rate-equation UCNP model, which represents a nonlinearity with memory, we also employed a simple memoryless nonlinearity model to provide physical intuition (see section S2 in the SI). This simplified model is useful for understanding how nonlinear frequency mixing depends on excitation power (Fig. S7), as well as on the relative amplitudes of the two excitation beams (section S2 in the SI). However, it neglects upconversion population dynamics and therefore cannot capture the frequency-dependent, low-pass response of UCNPs, more correctly described by the rate-equation model.

We next explored nanoparticle-engineering routes to increase the BFW of UCNPs. Motivated by the observed dependence of BFW (Fig. 3f) and SHW (Fig. 2g) on modulation frequency—indicative of photodynamic effects—we adopted a core-(multi)shell strategy to tune the photophysics of the UCNPs^16^. Specifically, we introduced a Yb^3+^-containing intermediate layer into the Tm^3+^-activated UCNPs, yielding NaYF_4_:20%Yb^3+^,0.5%Tm^3+^@NaYF_4_:20%Yb^3+^@NaYF_4_ (**YbTm@Yb@Y**) nanoparticles (Fig. S1). Under identical experimental conditions, the 800 nm emission from **YbTm@Yb@Y** UCNPs exhibited a substantially stronger BF component than from **YbTm@Y** (Fig. S8). We also synthesized Er^3+^-activated UCNPs with different structural architectures, including NaYF_4_:20%Yb^3+^,2%Er^3+^@NaYF_4_:20%Yb^3+^ (**YbEr@Yb**) and NaYF_4_:20%Yb^3+^,2%Er^3+^@NaYF_4_:20%Yb^3+^@NaYF_4_:30%Nd^3+^,20%Yb^3+^ (**YbEr@Yb@YbNd**) nanoparticles (Fig. S2), and evaluated their responses to superimposed modulated excitation. Across these designs, certain architectures produced stronger BF signals than **YbEr@Y** (Fig. S9).

The BFW of **YbEr@Yb@YbNd** UCNPs remained relatively insensitive to changes in the average base modulation frequency (Fig. S9b). Similarly, combining 980 nm (absorbed by Yb^3+^) and 808 nm (absorbed by Nd^3+^) laser excitation, modulated at different frequencies with a fixed difference frequency also produced robust and significant BF signals (Fig. S9d).

In the ${I}_{{\rm{exc}}}$ dependence **YbTm@Yb@Y** UCNPs exhibit a slightly higher overall nonlinearity under continuous-wave (CW) excitation compared to **YbTm@Y** (Figs. S10–S11). **YbEr@Yb@YbNd** UCNPs exhibit obviously lower overall steady-state nonlinearity than its simpler counterparts (Figs. S12–S14). Taken together, these results indicate that the overall steady-state nonlinearity of UCNPs is not the sole predictor of BF strength in multi-shell nanoparticles. In the architectures studied here, Yb^3+^ ions distributed across layers absorb excitation light and contribute via energy migration followed by energy transfer to Tm^3+^ or Er^3+^, providing multiple kinetic pathways through which dual-frequency excitation can couple into the emission. This coupling, rather than a single global power-law exponent, appears to govern BF strength (Fig. 3).

Importantly, these empirical trends should not be interpreted as implying that “more complex” necessarily means “better”. Performance depends on how specific design elements modify transfer rates, saturation behavior, and kinetics to favor the generation and transfer of modulation components. While the precise mechanistic attribution is not yet fully resolved and our discussion is necessarily qualitative, a quantitative link between composition/architecture and BF generation could be developed with advanced models, such as microscopic rate-equation approaches^17,18^, and random-walk models^19,20^, that explicitly account for ion distributions, interionic interactions, and full upconversion kinetics. Such analysis would provide a predictive framework for materials design.

### Suppression of laser residuals and ambient light in surface imaging via photophysical lock-in detection

We see significant potential in exploiting the BF signal for practical applications. In contrast to the SH signal, it can be generated at a sufficiently low arbitrary frequency, making it detectable also by low-speed detectors. Next, we explored this potential of the PP-LID approach, for background-free imaging of the BF signal from UCNPs using a frame-rate-limited sCMOS camera. In the imaging experiments, we used dual-frequency modulated excitation, using the setup schematically shown in Fig. S5, and recorded image sequences with an sCMOS camera at certain frame rates.

**Fig. 4:**
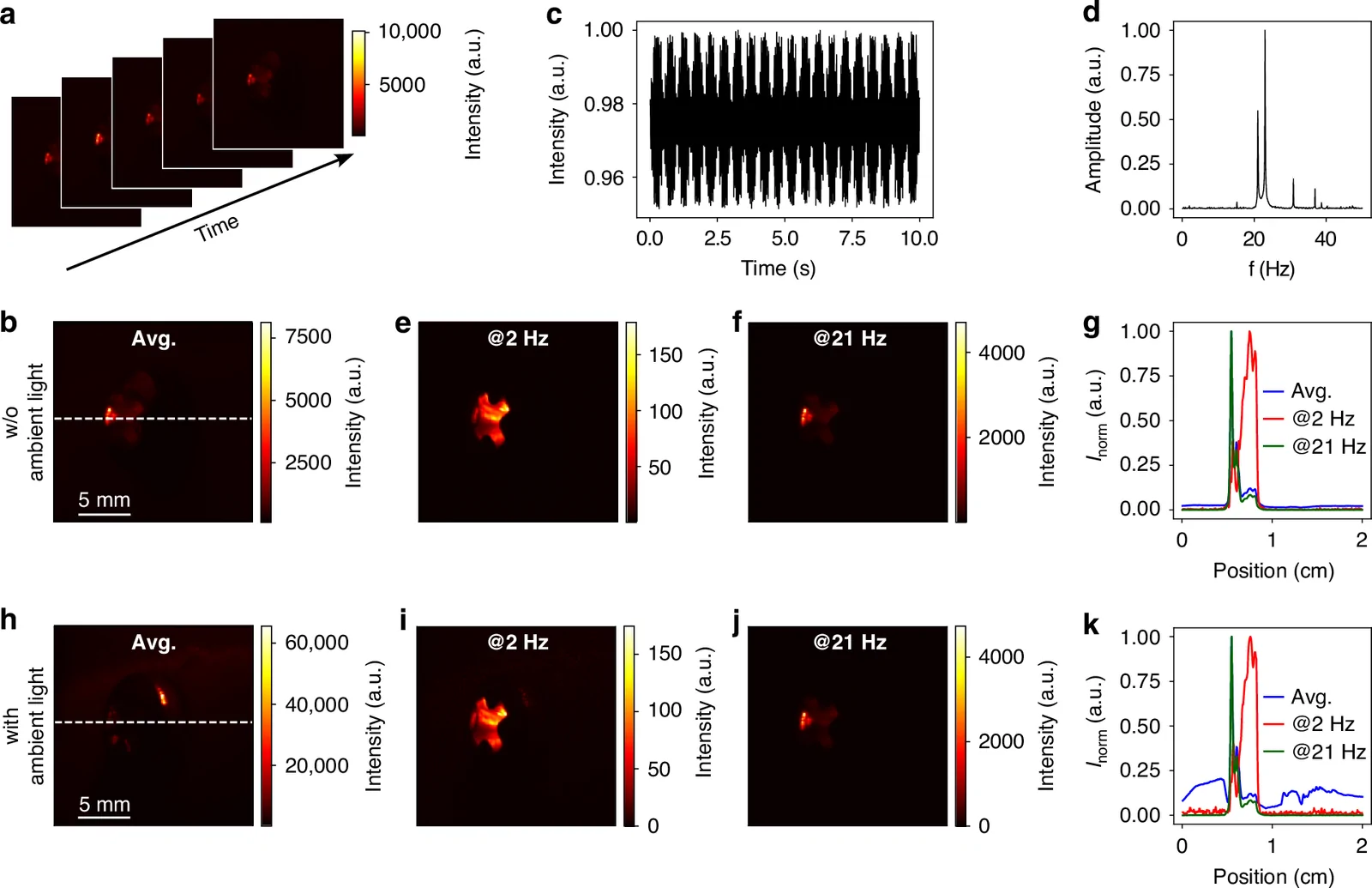
Wide-field upconversion luminescence (UCL) imaging of a surface object. **a** Image sequence of a **YbEr@Yb@YbNd** UCNP-capped screw cap under dual square-wave excitation of two 980 nm laser beams (excitation intensity: 210 W cm^−2^, each laser beam). **b** The averaged image of the UCNP-capped screw cap obtained from the recorded image sequence. **c** Time trace of the intensity, given by the integrated intensities in each of the images in the image sequence. **d** The FFT spectrum of the time trace of integrated signals in (**b**). **e** The BF (2 Hz)-filtered image of the screw cap. **f** The 21 Hz-filtered image of the screw cap. **g** Emission profiles along the marked line in the averaged, 2 Hz-filtered, and 21 Hz-filtered images. **h** The averaged image of the UCNP-capped screw cap of the image sequence recorded with nonuniform overwhelming ambient light but kept below detector saturation. **i** The BF (2 Hz)-filtered image of the screw cap of the image sequence recorded with ambient light. **j** The 21 Hz-filtered image of the screw cap of the image sequence recorded with ambient light. Intensities between images **(b**, **e**, **f**, **h**, **i**, **j)** can be compared. **k** Emission profiles along the marked line in the averaged, 21 Hz-filtered and 2 Hz-filtered images

We first conducted surface imaging experiments of a fluted ‘+’ pattern (a screw cap covered with **YbEr@Yb@YbNd** UCNPs, Fig. S15), using two 980 nm overlapped laser beams, modulated at 21 and 23 Hz for excitation (Fig. 4a). The averaged image (over all acquired images in the sequence) largely displays light collected from the surrounding region of the fluted ‘+’ pattern (Fig. 4b), indicating substantial surface reflection of laser light despite the use of a 950 nm short-pass filter. Next, we analyzed the data in the frequency domain. For each image in the sequence, the emission intensity was integrated across the entire field, yielding summed intensity time-traces clearly reflecting the excitation modulation (Fig. 4c). FFT analysis of these time-traces then revealed the BF signal at 2 Hz, alongside signals at the two base modulation frequencies (Fig. 4d). We then frequency-filtered the 2 Hz signal in the image sequence for each pixel and reconstructed the image (detailed in Methods). The prominent residual excitation light present in the averaged image (Fig. 4b) is almost entirely suppressed in this BF image, while the UCNP signal pattern is preserved (Fig. 4e). This effect is further highlighted in emission profiles across the fluted region (Fig. 4g). In contrast, filtering at either of the base modulation frequencies followed by image reconstruction does not achieve the same effect (Fig. 4f, Fig. S16). To further stress the experimental conditions, we then introduced nonuniform overwhelming ambient light but kept below detector saturation during the measurements. In the averaged image (Fig. 4h), the UCL signal pattern as well as the laser light reflection was then entirely obscured by the ambient light. However, the frequency-filtering approach at 2 Hz could successfully recover the UCL signal pattern (Fig. 4i), but not at 21 Hz (Fig. 4j, k).

### Background-free deep-tissue upconversion imaging enabled by photophysical lock-in detection

**Fig. 5:**
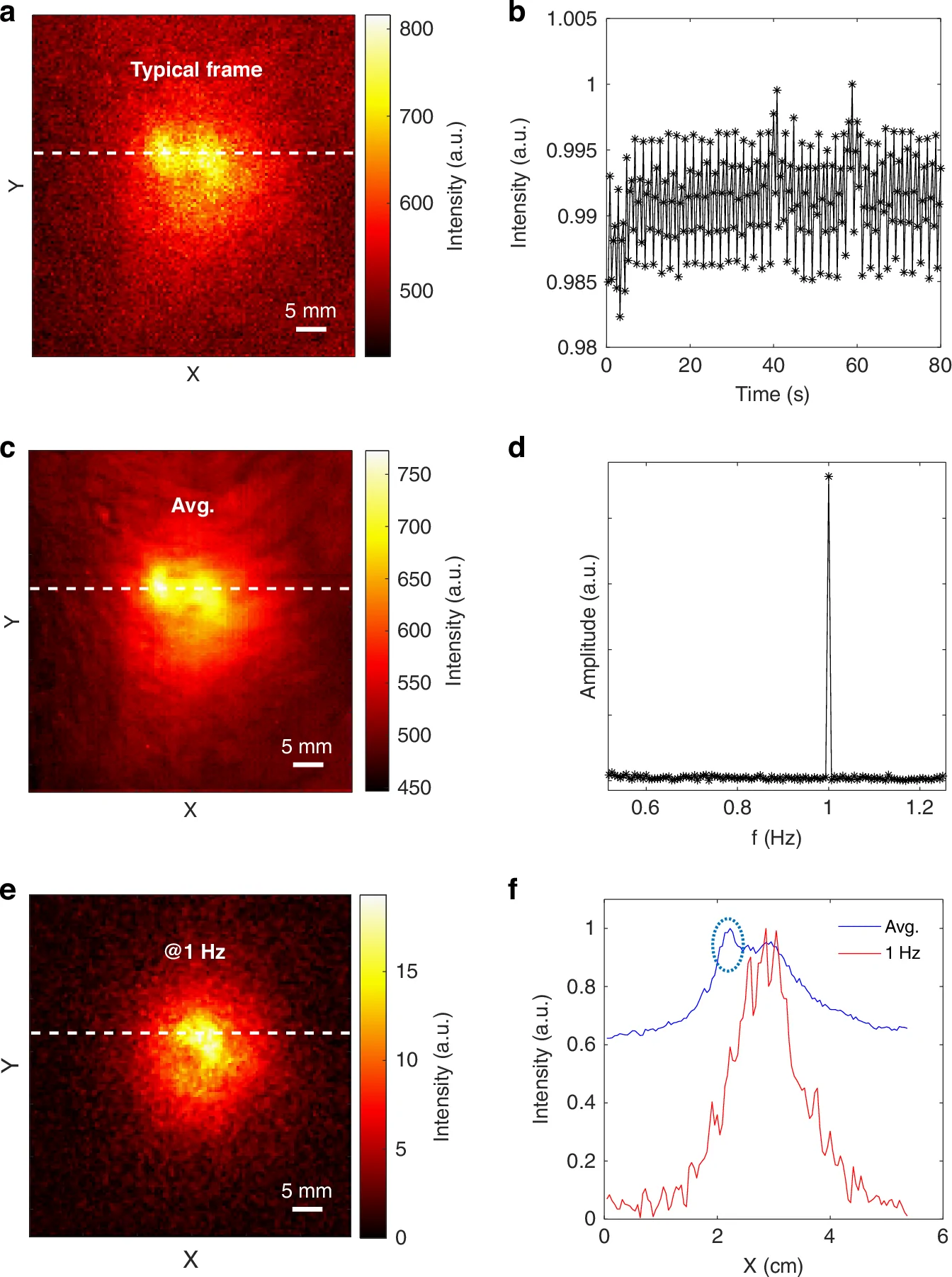
Diffuse optical imaging of a UCNP inclusion in deep tissue. **a** A typical frame in the image sequence (video 1) of a capillary filled with **YbTm@Yb@Y** (10 mg mL^−1^) embedded in chicken breast tissue at an approximate depth of 5 mm under dual square-wave excitation of two 980 nm laser beams, measured in presence of a constant ambient light level. Time-averaged excitation intensity: 35 mW cm^−2^, for each laser beam. Frame exposure time: 400 ms. **b** Time trace of integrated intensity values from the image sequence in video 1. **c** Averaged image of the sample (UCNP-filled capillary behind chicken tissue), obtained from the recorded image sequence in video 1. **d** The FFT spectrum of the time trace of integrated signals in (**b**). **e** The BF (1 Hz)-filtered image. **f** Emission profiles along the marked lines in the averaged (**c**) and 1 Hz-filtered **e** images, normalized to their own maximum values. The blue dashed ellipse in (**f**) marks the laser light contaminated pattern

We next conducted tissue imaging experiments to evaluate the performance of the PP-LID approach under biologically relevant conditions. A capillary tube (1.5 mm diameter) filled with **YbTm@Yb@Y** suspension (10.0 mg mL^−1^) was positioned behind a 5 mm-thick slab of chicken breast tissue, simulating light scattering and attenuation in biological tissue. To excite UCL, two co-axial laser beams (980 nm, modulated at 22 Hz and 23 Hz, both with 50% duty cycle) irradiated the tissue surface at an incident angle of ~45°, while the camera collected emission signals from a similar angle relative to the normal surface (Fig. S17). To create a challenging imaging scenario, the total time-averaged ${I}_{{\rm{exc}}}$ was maintained at approximately 72 mW cm^−2^—nearly an order of magnitude below the ANSI safety limit for continuous-wave 980 nm skin exposure (~710 mW cm^−2^)^21^. A 950 nm long-pass cleanup filter in the excitation path suppressed any residual visible laser emission (Fig. S18), and emission filters (two 950-nm short-pass filters (FF01-950/SP-25, OD6, Semrock) in combination with a 810-nm band-pass filter (ET810/90, OD6, Chroma)) were used in the detection path to clean the UCL signal near 800 nm. Image sequences were recorded with a frame exposure time of 400 ms, chosen to accommodate the low signal level under the weak excitation and deep imaging conditions while still capturing the expected BF signal at 1 Hz. A representative frame, out of the entire sequence (video 1), and the average of all frames are presented in Fig. 5a, c, respectively. As anticipated, the capillary shape was not sharply resolved due to significant light scattering through the tissue. Frame averaging did not appreciably improve the SBR, due to the weak UCL signal and the relatively deep embedding of the capillary. To extract the periodic BF signal, the summed intensity across frames was analyzed over time (Fig. 5b). Subsequent FFT analysis clearly identified the 1 Hz BF component (Fig. 5d). Comparison of the averaged image (Fig. 5c) and the BF-filtered image (Fig. 5e) shows that the significant excitation-light leakage present in the former —despite the use of a nominal OD of 18 to block the excitation light (Fig. S5) —is effectively suppressed in the latter, together with other substantial background contributions. This conclusion is supported by spectral measurements of the light transmitted through the detection filters (Fig. S19), which clearly reveal residual laser leakage. Additional spectral control measurements, performed with the emission filters in place but without UCNP inclusion while the excitation lasers remained on, further confirm that the leakage is confined to the excitation wavelength region around 980 nm and is absent at shorter wavelengths (Fig. S20). Profile analyses along the dashed line in Fig. 5c, e demonstrate that the SBR was improved by nearly an order of magnitude using the PP-LID approach (Fig. 5f). Also with dynamically varying ambient light our PP-LID approach could successfully detect the 1 Hz BF signal (Fig. S21), suppressing both excitation leakage and ambient light fluctuations, and improving the SBR by approximately one order of magnitude (video 2, Fig. S21f).

Subsequently, we reduced the concentration of **YbTm@Yb@Y** UCNPs in the capillary down to 100 µg mL^−1^. To address the weak UCL signal we increased the camera exposure time and correspondingly designated the BF signal at 0.1 Hz (base modulation frequencies at 22 and 22.1 Hz). The PP-LID approach could still image the UCNP inclusion well through a 2-mm thick chicken tissue with a high SBR using a total time-averaged ${I}_{{\rm{exc}}}$ ~ 510 m Wcm^−2^, while eliminating the deleterious effect of laser light leakage (Fig. S22).

For deep tissue imaging, it is worth emphasizing that PP-LID primarily improves SBR and contrast; it does not alter the fundamental spatial bandwidth set by diffusive light transport. In wide-field luminescence diffuse optical tomography (LDOT)-type geometries^22,23^, the achievable spatial resolution is governed by the tissue’s optical properties (scattering and absorption), the depth of the UCNP inclusion, and the sampling and reconstruction scheme, and is therefore typically on the millimeter scale. PP-LID can sharpen reconstructions indirectly—by enabling less aggressive regularization and better discrimination of nonlinear components—but it does not circumvent the scattering limit or permit a single sample-independent numeric resolution.

Within this context, our simulated data indicate that BFWs tend to decrease with higher ${I}_{{\rm{exc}}}$ but remain close to constant at low ${I}_{{\rm{exc}}}$ (Fig. 1p, s). Within the ANSI safety limit^21^, upconversion saturation is then unlikely, and the UCNPs retain a high nonlinearity, as confirmed in our measurements (Fig. S23). From a signal-to-background point of view, lower (tissue attenuated) excitation intensities are thus not a complication per se. Moreover the enhanced SBR provided by our PP-LID approach gives better prerequisites for LDOT^22^ image reconstruction. Finally, our method is not inherently reliant on the superposition of two separate laser beams; a practical and scalable implementation is to use a single laser beam with modulated excitation that combines two modulation waveforms.

## Discussion

UCNPs can function as frequency mixers in response to a modulated excitation intensity. Specifically, when exposed to sinusoidal or square-wave excitation, UCNPs can generate new frequency components that are not contained in the input excitation function. For modulated excitation containing two base modulation frequencies, they act as frequency mixers, producing beat-frequency (BF), as well as sum-frequency signals. The generation of the BF signal stems from the inherent nonlinear response of UCNPs to excitation intensity and can be enhanced significantly by manipulating the complexity of upconversion pathways via nanoparticle core-(multi)shell engineering. It is important to note that the proposed PP-LID approach is fundamentally distinct from previously reported frequency modulation techniques using UCNPs^24^, which employed conventional modulation and lock-in detection at the same frequency. Through selective BF signal detection, PP-LID provides an orthogonal approach to, and may be used in combination with, widely used background-suppression methods, such as time-gated detection^25,26^. As a standalone approach, PP-LID requires no precise synchronization between the excitation source and detector, is insensitive to ambient light interference provided detector saturation is avoided, and does not impose stringent requirements on detector time resolution. Future work will focus on systematically investigating the impact of nanocrystal architecture, including ion migration and surface-related nonradiative processes, to further enhance the brightness achievable with PP-LID and to develop more quantitative models for practical applications. For in vivo applications, physiological motion (e.g., breathing or blood flow) may introduce temporal fluctuations that could affect the stability of the extracted BF signals. Addressing such effects through faster acquisition or motion correction strategies will also be an important direction for future work.

## Materials and methods

### Synthesis of nanoparticles

#### Chemicals and Materials

Anhydrous yttrium(III), ytterbium(III), neodymium(III), erbium(III), and thulium(III) chlorides (99.9%), oleic acid (90%), octadec-1-ene (90%), methanol, sodium hydroxide, ammonium fluoride were purchased from Sigma-Aldrich (St. Louis, MO, USA). Cyclohexane was purchased from VWR (Radnor, PA, USA).

#### Synthesis of NaYF_4_:Yb^3+^,Tm^3+^ and NaYF_4_:Yb^3+^,Er^3+^ core nanoparticles

The core nanoparticles were synthesized according to previously reported protocols^27,28^. Into a 100-mL three-neck round-bottom flask, 1 mmol of lanthanide chlorides, oleic acid (6 mL), and octadec-1-ene (15 mL) were mixed. To prepare NaYF_4_:Yb^3+^,Er^3+^, 0.78 mmol YCl_3_, 0.2 mmol YbCl_3_, and 0.02 mmol ErCl_3_ were used. To prepare NaYF_4_:Yb^3+^,Tm^3+^, 0.795 mmol YCl_3_, 0.2 mmol YbCl_3_ and 0.01 mmol TmCl_3_ were used. In each case, the mixture was heated at 160 °C for 30 min with stirring under continuous argon flow until the mixture became homogeneous, and then it was cooled down to room temperature. After cooling, a methanolic solution of NaOH (2.5 mmol) and NH_4_F (4 mmol) was added to the reaction mixture. The reaction temperature gradually increased from room temperature to 300 °C. Different reaction time was used to prepare different nanoparticles. Next, the mixture was naturally cooled down to room temperature, and the NaYF_4_:Yb^3+^,Er^3+^ or NaYF_4_:Yb^3+^,Tm^3+^ nanoparticles were collected by centrifugation. After centrifugation, the solution above a white pellet (precipitate of nanoparticles) was discarded and the nanoparticles were dispersed in cyclohexane (10 mL), precipitated by ethanol (5 mL), and centrifugated. This step was repeated one more time and, finally, the nanoparticles were dispersed in cyclohexane for subsequent use.

#### Synthesis of NaYF_4_:Yb^3+^,Tm^3+^@NaYF_4_:x%Yb^3+^ and NaYF_4_:Yb^3+^,Er^3+^@NaYF_4_:x%Yb^3+^ core-shell nanoparticles

Core-shell NaYF_4_:Yb^3+^,Er^3+^@NaYF_4_:x%Yb^3+^ and NaYF_4_:Yb^3+^,Tm^3+^@NaYF_4_:x%Yb^3+^ nanoparticles were synthesized according to previously reported protocols with modifications^27,28^. Stoichiometric lanthanide chloride shell precursors, oleic acid (6 mL), and octadec-1-ene (15 mL) were mixed and heated at 160 °C for 30 min under argon flow to get a homogeneous yellowish solution and cooled down. After cooling, the suspension of pre-synthesized core nanoparticles, a methanolic solution of NaOH (1.25 mmol) and NH_4_F (2 mmol) were added to the reaction mixture and heated gradually to 300 °C for 1 h. The mixture was cooled down to room temperature and the core-shell nanoparticles were collected by centrifugation. After centrifugation, the top solution was discarded and the core-shell nanoparticles left were dispersed in cyclohexane (10 mL), precipitated by ethanol (5 mL), and centrifugated. This step was repeated one more time and, finally, the core-shell nanoparticles were dispersed in cyclohexane for subsequent use.

#### Synthesis of NaYF_4_:20%Yb^3+^,0.5%Tm^3+^@NaYF_4_:20%Yb^3+^@NaYF_4_ and NaYF_4_:20%Yb^3+^, 2%Er^3+^@NaYF_4_:20%Yb^3+^@NaYF_4_:30%Nd^3+^, 20%Yb^3+^ core-shell-shell nanoparticles

NaYF_4_:20%Yb^3+^,0.5%Tm^3+^@NaYF_4_:20%Yb^3+^ nanoparticles were first synthesized and then used as the seed nanoparticles to grow a NaYF_4_ layer, yielding NaYF_4_:20%Yb^3+^,0.5%Tm^3+^@NaYF_4_:20%Yb^3+^@NaYF_4_, following the procedure described above.

NaYF_4_:20%Yb^3+^, 2%Er^3+^@NaYF_4_:20%Yb^3+^ nanoparticles were first synthesized and then used as the seed nanoparticles to grow a NaYF_4_:30%Nd^3+^, 20%Yb^3+^ layer, yielding NaYF_4_:20%Yb^3+^, 2%Er^3+^@NaYF_4_:20%Yb^3+^@NaYF_4_:30%Nd^3+^, 20%Yb^3+^ UCNPs, following the procedure described above.

### Characterization

TEM micrographs of nanoparticles were acquired by a Talos 120 C G2 transmission electron microscope (Thermo Fisher Scientific). The phase structures of the nanoparticles were characterized by X-ray diffraction. Upconversion emission spectra were measured on an Einburgh Instruments FS5spectrofluorometer or an Ocean Optics QEPro spectrometer under excitation of 980-nm diode lasers.

### Simulations of excitation modulation response of linear fluorophores

The electronic state population dynamics of a linear fluorophore, as depicted in the Jablonski diagram in Fig. 1a, are modeled by the following rate equations:

3 $$\frac{{\rm{d}}[{{\rm{S}}}_{0}]}{{\rm{d}}t}={-k}_{01}P(t)\left[{{\rm{S}}}_{0}\right]+{k}_{10}\left[{{\rm{S}}}_{1}\right]+{k}_{{\rm{T}}}\left[{{\rm{T}}}_{1}\right]$$

4 $$\frac{{\rm{d}}\left[{{\rm{S}}}_{1}\right]}{{\rm{d}}t}={k}_{01}P(t)\left[{{\rm{S}}}_{0}\right]-{k}_{10}\left[{{\rm{S}}}_{1}\right]-{k}_{{\rm{isc}}}\left[{{\rm{S}}}_{1}\right]$$

5 $$\frac{{\rm{d}}\left[{{\rm{T}}}_{1}\right]}{{\rm{d}}t}={k}_{{\rm{isc}}}\left[{{\rm{S}}}_{1}\right]-{k}_{{\rm{T}}}\left[{{\rm{T}}}_{1}\right]$$

Here, $[{{\rm{S}}}_{0}]$, $\left[{{\rm{S}}}_{1}\right]$, and $\left[{{\rm{T}}}_{1}\right]$ denote the population probabilities of the singlet ground state (${{\rm{S}}}_{0}$), the singlet excited state (${{\rm{S}}}_{1}$), and the triplet state (${{\rm{T}}}_{1}$), respectively; ${k}_{01}$ is the peak excitation rate, ${k}_{10}$ is the radiative decay rate of the ${{\rm{S}}}_{1}$ state, ${k}_{{\rm{isc}}}$ is the inter-system crossing rate, and ${k}_{{\rm{T}}}$ is the decay rate of the ${{\rm{T}}}_{1}$ state; P(t) represents the peak-normalized excitation function, which can take different forms, sinusoidal wave, or square wave, or their superpositions, in simulating the response of the fluorophore to different optical stimuli. The parameter values used in the simulations of Fig. 1c, e are summarized in Table 1. The Eqs. (3–5) were solved using an open-source software COPASI, using the initial conditions $\left[{{\rm{S}}}_{0}\right]\left(t=0\right)=1$, and $\left[{{\rm{S}}}_{1}\right]\left(t=0\right)=\left[{{\rm{T}}}_{1}\right]\left(t=0\right)=0$.

**Table 1:** Parameter values used in the simulations of excitation modulation response of linear fluorophores

${k}_{01}$ (s^-1^)	${k}_{10}$ (s^-1^)	${k}_{{\rm{isc}}}$ (s^-1^)	${k}_{{\rm{T}}}$ (s^-1^)
1.2 × 10^7^	2.5 × 10^8^	1 × 10^7^	5 × 10^5^

### Simulations of excitation modulation response of upconversion nanoparticles

Numerical simulations of the response of upconversion nanoparticles to modulated excitation were performed based on a simplified two-photon upconversion model depicted in Fig. 1f.

The kinetics of this upconversion system are modeled by the following rate equations:

6 $$\frac{{\rm{d}}{n}_{1}}{{\rm{d}}t}=-{W}_{51}{n}_{1}{n}_{5}+{k}_{21}{n}_{2}+{k}_{31}{n}_{3}$$

7 $$\frac{{\rm{d}}{n}_{2}}{{\rm{d}}t}={W}_{51}{n}_{1}{n}_{5}-{W}_{52}{n}_{2}{n}_{5}{-k}_{21}{n}_{2}$$

8 $$\frac{{\rm{d}}{n}_{3}}{{\rm{d}}t}={W}_{52}{n}_{2}{n}_{5}{-k}_{31}{n}_{3}$$

9 $$\frac{{\rm{d}}{n}_{4}}{{\rm{d}}t}=-{k}_{45}P(t){n}_{4}+{k}_{54}{n}_{5}+{W}_{51}{n}_{1}{n}_{5}+{W}_{52}{n}_{2}{n}_{5}$$

10 $$\frac{{\rm{d}}{n}_{5}}{{\rm{d}}t}={k}_{45}P(t){n}_{4}-{k}_{54}{n}_{5}-{W}_{51}{n}_{1}{n}_{5}-{W}_{52}{n}_{2}{n}_{5}$$

where *n*_*i*_ is the population density of energy state ***i*** *(****i*** = 1–5*)* in Fig. 1f; ${k}_{45}$ is the excitation pumping rate for the transition of state **4** → state **5;** ${k}_{54}$, ${k}_{21}$ and ${k}_{31}$ are the decay rates for state **5**, state **2**, and state **3**, respectively; ${W}_{51}$ and ${W}_{52}$ are the coefficients for ETU1 and ETU2 (see Fig. 1f), respectively; P(t) represents the peak-normalized excitation function, sinusoidal wave, or square wave, or their superpositions. The parameter values used for the simulations of UCNPs are summarized in Table 2.

**Table 2:** Parameter values used in the simulations of excitation modulation response of upconversion nanoparticles

${k}_{45}$ (s^-1^)	${k}_{54}$ (s^-1^)	${k}_{21}$ (s^-1^)	${k}_{31}$ (s^-1^)	${W}_{51}$(cm^3^ s^-1^)	${W}_{52}$ (cm^3^ s^-1^)
7.9	1 × 10^3^	7.6 × 10^2^	1.1 × 10^4^	2 × 10^−16^	2 × 10^−15^

The Eqs. (6–10) were solved using an open-source software COPASI through its python interface BasiCO^29,30^, using the initial conditions $\left[{{\rm{n}}}_{1}\right]\left(t=0\right)=1.25\times {10}^{20} \,{{\rm{cm}}}^{-3}$, $\left[{{\rm{n}}}_{4}\right]\left(t=0\right)=1.52\times {10}^{21} \,{{\rm{cm}}}^{-3}$, and $\left[{{\rm{n}}}_{2}\right]\left(t=0\right)=\left[{{\rm{n}}}_{3}\right]\left(t=0\right)=\left[{{\rm{n}}}_{5}\right]\left(t=0\right)=0 {\,{\rm{cm}}}^{-3}$.

### Temporal demodulation of image sequences

In the imaging experiments, we used dual-frequency modulated excitation, and recorded image sequences with an sCMOS camera at certain frame rates, referring to videos 1 and 2 for example. In the image analysis, global time traces were obtained by integrating the intensity over each frame in the sequence, and an FFT was then applied to these traces to analyze the frequency components.

In obtaining a target-frequency-filtered image, the processing is temporal:

- Extract the time series $I(x,y,{t}_{n})$ for each pixel.
- For a target frequency ${f}_{0}$ (e.g., the BF or the base frequencies), compute per-pixel Fourier amplitude via an FFT or equivalently a lock-in operation:
  11 $$A\left(x,y;{f}_{0}\right)=\sqrt{{{\langle }}I(x,y,t)\cos 2\pi {f}_{0}t{{{\rangle }}}^{2}+{{\langle }}I(x,y,t)\sin 2\pi {f}_{0}t{{{\rangle }}}^{2}}$$
- Render the amplitude map $A(x,{y;}{f}_{0})$ as the “filtered” image at ${f}_{0}$. BF images use Δf.

## Supplementary information

Supporting Information

video 1

video 2

## Data Availability

All relevant raw data behind this study are available via https://doi.org/10.5281/zenodo.15784536.
