# Supplementary material for: Photophysical lock-in detection enables background-free upconversion emission imaging
Source: Light Sci Appl. 2026 Sep 7;15:371. doi: 10.1038/s41377-026-02414-2 (PMC13550578; doi:10.1038/s41377-026-02414-2)
Supplement: Supplementary file 1 [file 41377_2026_2414_MOESM1_ESM.docx]

**Supplementary Information**

**Photophysical lock-in detection enables background-free upconversion emission imaging**

Niusha Bagheri^a^, Chenyi Wang^b^, Du Guo^a^, Anbharasi Lakshmanan^a^, Qi Zhu^a^, Xu Chen^a^, Nahid Ghazyani^e^, Qiuqiang Zhan^b^, Georgios A. Sotiriou^c,d^, Haichun Liu*^a^, Jerker Widengren*^a^

*^a^ Experimental Biomolecular Physics, Department of Applied Physics, KTH Royal Institute of Technology, SE-106 91, Stockholm, Sweden.*

*^b^ Centre for Optical and Electromagnetic Research, South China Academy of Advanced Optoelectronics, South China Normal University, Guangzhou 510006, China.*

*^c^ Department of Microbiology Tumor and Cell Biology Karolinska Institute, SE-171 77, Stockholm, Sweden.*

*^d^ Department of Chemistry, Science for Life Laboratory (SciLifeLab), Stockholm University, SE-114 18, Stockholm, Sweden.*

*^e^ Faculty of Physics, Kharazmi University, Tehran, Iran.*

**Contents:**

**Section S1**. **Duty-cycle considerations and Fourier analysis**

**Section S2**. **Memoryless nonlinearity model for intuitive analysis of frequency mixing**

**Supplementary Figures S1-S23**

**Section S1**. **Duty-cycle considerations and Fourier analysis**

We model a square-wave modulation $s_{D}(t)$ of period $T=1/f$ and duty cycle $D\in(0, 1)$, and amplitude varying between 0 and $V_{\max}$. Its Fourier series is

$$s_{D}\left( t \right)=V_{\max}D+\sum_{k=1}^{\infty} \frac{2V_{\max}}{\pi k}\sin\left( \pi kD \right)\cos\left( 2\pi kft-\pi kD \right) \left( S1 \right).$$

Thus, the amplitude of the $k$-th harmonic scales as

$C_{k}=\frac{2V_{\max}}{\pi k}\left| \sin\left( \pi kD \right) \right|$	(S2)

i.e., as $\left| \sin\left( \pi kD \right) \right|/k$. At $D=0.5$,

$\sin(\pi k/2)=0$	(S3)

for even $k$, so all even harmonics of the excitation are identically zero; only odd harmonics remain. Consequently, any even-harmonic content observed in the emission cannot originate from linear bleed-through of the excitation and instead requires a nonlinear generation mechanism.

For two square-wave excitations at $f_{1}$ and $f_{2}$, both with $D=0.5$, the excitation spectrum contains the carrier frequencies and their odd harmonics but no component at the beat frequency (BF) $\Delta f=\mid f_{1}-f_{2}\mid$. Therefore, an emission line at $\Delta f$ provides evidence of nonlinear frequency mixing in the UCNPs.

Finally, for a fixed peak drive $V_{\max}$, $D=0.5$ maximizes the amplitude for the fundamental harmonic, yielding

$C_{1}=\frac{2V_{\max}}{\pi}$	(S4)

Although a unipolar square wave retains a nonzero DC component $V_{\max}D$, choosing $D=0.5$suppresses all even harmonics, facilitating consistent signal-to-background ratio (SBR) comparisons across datasets.

**Section S2**. **Memoryless nonlinearity model for intuitive analysis of frequency mixing**

To provide some physical intuition complementary to the rate-equation modeling, we consider a simplified memoryless nonlinearity model describing the luminescence response to modulated excitation. In this model, the upconversion emission is approximated as an instantaneous nonlinear function of the excitation intensity, neglecting population dynamics and finite excited-state lifetimes. While this approximation does not aim to quantitatively reproduce the photophysics of UCNPs, it is useful for developing intuition about nonlinear frequency mixing under modulated excitation. Specifically, we model the excitation as the superposition of two sinusoidally modulated beams with frequencies $f_{1}$ and $f_{2}$,

$$X\left( t \right)=A_{1}\left[ \sin\left( {2\pi f}_{1}t \right)+1 \right]+A_{2}\left[ \sin\left( {2\pi f}_{2}t \right)+1 \right] \left( S5 \right)$$

where $A_{1}$ and $A_{2}$ denote the modulation amplitudes of the two excitation components. The additive offset ensures that the excitation intensity remains non-negative. The emitted signal is then described by a power-law nonlinearity,

$$Y(t)=X(t)^{n} \left( S6 \right)$$

where the exponent $n$ represents an effective nonlinearity extracted from the excitation power dependence of the steady-state upconversion emission (see Fig. 1k in the main text).

**S2.1 Impact of Effective Nonlinearity** $n$

Within this framework, nonlinear frequency mixing arises naturally as a mathematical consequence of the nonlinear mapping between excitation and emission. Expansion of $X(t)^{n}$ generates frequency components at harmonics of $f_{1}$ and $f_{2}$ , as well as at their sum and difference (i.e., the BF signal at $\mid f_{1}-f_{2}\mid$) frequencies. The relative amplitudes of these components depend on both the effective nonlinearity $n$ and the relative excitation amplitudes $A_{1}$ and $A_{2}$. As a result, this model provides qualitative insight into how increasing excitation power (reflected in a larger effective $n$) or balancing the two excitation amplitudes enhances the strength of frequency-mixed signals.

Figure S7 shows the calculated beat-frequency weight (BFW), defined per equation (2) in the main text, as a function of excitation intensity using the memoryless nonlinearity model. The model predicts a monotonic decrease of BFW with excitation intensity, reflecting the decrease of the effective nonlinearity exponent $n$. This trend is qualitatively consistent with the results obtained from the rate-equation simulations presented in the main text (Fig. 1p), confirming that stronger nonlinear excitation–emission coupling enhances frequency mixing.

However, a quantitative comparison reveals that the memoryless model systematically overestimates the BFW values relative to those obtained from the rate-equation model (Figure S7, Fig. 1p). This discrepancy arises from the intrinsic assumptions of the model. By treating the nonlinear response as instantaneous, the memoryless model implicitly assumes that all frequency components generated by the nonlinearity are transmitted without dynamic attenuation. In contrast, the rate-equation model accounts for the finite lifetimes of the relevant excited states, which introduce a frequency-dependent response that suppresses the modulation depth of the emission. As a result, the amplitudes of both the carrier-frequency and BF components are dynamically filtered, leading to reduced BFW values. This comparison highlights that the memoryless model captures the qualitative scaling of frequency mixing with excitation power but overestimates its magnitude by neglecting population dynamics.

**S2.2 Impact of Relative Excitation Amplitudes** $\boldsymbol{A}_{\boldsymbol{1}}$ **and** $\boldsymbol{A}_{\boldsymbol{2}}$

The memoryless nonlinearity model can also provide intuition for how the excitation amplitudes $A_{1}$ and $A_{2}$ influence the BFW. Importantly, in UCNPs the effective nonlinearity exponent $n$ is not a fixed constant, but depends on the excitation level due to upconversion saturation. Therefore, amplitude changes affect BFW through two coupled mechanisms: (i) the instantaneous mixing at fixed $n$, and (ii) the indirect change of $n$ with the total excitation intensity.

To make this explicit, we consider the excitation and emitted signal specified in equations (S5) and (S6), respectively, with $n(I_{\mathrm{avg}})$ the effective power-law exponent evaluated at the relevant average excitation level $I_{\mathrm{avg}}$ (in practice set by the DC component of $X(t)$, which scales with $A_{1}+A_{2}$).

***Common scaling of both amplitudes***

In an idealized memoryless nonlinearity with *constant* exponent $n$, scaling both amplitudes by a common factor $k$ (i.e., $A_{1}\to kA_{1}$, $A_{2}\to kA_{2}$) leads to $X(t)\to kX(t)$ and hence $Y(t)\to k^{n}Y(t)$. In that case, all Fourier components of $Y(t)$ scale by the same factor $k^{n}$, and the BFW—defined as a ratio of Fourier magnitudes—would be invariant under common amplitude scaling.

However, for UCNPs the effective exponent typically decreases with increasing excitation intensity, reflecting upconversion saturation. Under common scaling, the increase of the DC excitation level ($\propto A_{1}+A_{2}$) therefore leads to a reduced $n(I_{\mathrm{avg}})$, which partially counteracts the tendency of a stronger nonlinearity to enhance frequency mixing. Consequently, when $n$ is treated as intensity-dependent, BFW is generally not strictly scale-invariant: it can vary with the overall excitation level through the implicit dependence $n(I_{\mathrm{avg}})$.

***Changing the amplitude balance*** $\boldsymbol{A}_{\boldsymbol{1}}\boldsymbol{/}\boldsymbol{A}_{\boldsymbol{2}}$

Varying the relative balance between $A_{1}$ and $A_{2}$ affects BFW in two ways. At fixed $n$, the BF component at $\mid f_{1}-f_{2}\mid$ originates from nonlinear cross terms involving both frequencies and is therefore strongest when both excitation components are present with comparable amplitudes. This can be illustrated for $n=2$ by writing (for notational simplicity) $X(t)=C+A_{1}\sin(2\pi f_{1}t)+A_{2}\sin(2\pi f_{2}t)$ with $C\approx A_{1}+A_{2}$. The difference-frequency term arises from the cross product $2A_{1}A_{2}\sin(2\pi f_{1}t)\sin(2\pi f_{2}t)$, giving a component at $\mid f_{1}-f_{2}\mid$ proportional to $A_{1}A_{2}$, whereas the carrier components scale primarily with $CA_{1}$ and $CA_{2}$. With the present definition of BFW, this yields the approximate scaling

$$\mathrm{BFW}\approx\frac{A_{1}A_{2}}{\left( A_{1} + A_{2})^{2} \right.} \left( n=2 \right) (S7)$$

which is maximized near $A_{1}\simeq A_{2}$ and tends to zero when one component dominates.

In UCNPs, however, changing $A_{1}/A_{2}$ can also change the total excitation level (and thus $n$) unless $A_{1}+A_{2}$ is held constant. Even when $A_{1}+A_{2}$ is held fixed, changing the ratio $A_{1}/A_{2}$ modifies the instantaneous excitation waveform and therefore the distribution of excitation intensities sampled over time. If effective nonlinearity depends on the instantaneous excitation intensity, such changes can shift the effective value of $n$ governing the frequency-mixing response.

Overall, the simplified memoryless nonlinearity model predicts that balanced excitation amplitudes are favorable for enhancing BF signals, but it also highlights that the strength of frequency mixing in UCNPs is modulated by the excitation-dependent nonlinearity $n(I)$. Quantitative predictions therefore require accounting for how $n$ evolves with excitation level, and ultimately benefit from the dynamic rate-equation framework used in the main text.

**Supplementary figures**

**Figure S1** Transmission electron microscopic (TEM) images of **(a)** NaYF_4_:20%Yb^3+^, 0.5%Tm^3+^@NaYF_4_ (**YbTm@Y**) and **(b)** NaYF_4_:20%Yb^3+^,0.5%Tm^3+^@NaYF_4_:20%Yb^3+^@NaYF_4_ (**YbTm@Yb@Y**) UCNPs. **(c)** X-ray diffraction (XRD) patterns of **YbTm@Y** and **YbTm@Yb@Y UCNPs.**

**Figure S2** TEM images of NaYF_4_:20%Yb^3+^, 2%Er^3+^@NaYF_4_ (**YbEr@Y**), NaYF_4_:20%Yb^3+^,2%Er^3+^@NaYF_4_:20%Yb^3+^ (**YbEr@Yb**)**,** and NaYF_4_:20%Yb^3+^, 2%Er^3+^@NaYF_4_:20%Yb^3+^@NaYF_4_:30%Nd^3+^, 20%Yb^3+^ (**YbEr@Yb@YbNd)** UCNPs.

**Figure S3 Optical setup to investigate the response of UCNPs to a single-beam square-wave excitation with a 50% duty cycle.** Herein, a continuous-wave (CW) laser at 980 nm was used as the excitation source, and a mechanical chopper modulated the excitation light to generate the desired square wave. The laser beam was split into two paths by a cube beamsplitter: one path was monitored by a photodiode to track the excitation wave, while the other path illuminated the UCNP sample, generating upconversion emissions that were detected by an avalanche photodiode (APD). The chopper allowed us to vary the modulation frequency.

**Figure S4 Second-harmonic (SH) signal generation of NaYF_4_:20%Yb^3+^, 2%Er^3+^@NaYF_4_ (YbEr@Y) UCNPs under square-wave excitation.** **(a)** Upconversion nanoparticle structure (Left) and population mechanism (Right) of the studied **YbEr@Y** UCNPs **(b)** The applied square-wave excitation with a 50% duty cycle (maximum excitation intensity: 20.3 W cm^-2^) and **(c)** its magnitude spectrum of the fast Fourier transform (FFT). **(d)** The generated upconversion emission (500-700 nm) and **(e)** its FFT magnitude spectrum. The dependence of the SH weight (SHW) of the upconversion emission (500-700 nm) on **(f)** excitation modulation frequency and **(g)** excitation intensity.

**Figure S5 Optical setup to investigate the response of UCNPs to dual beam square-wave excitation both with a 50% duty cycle.** Herein, two continuous-wave (CW) lasers at selected wavelengths (808 or 980 nm) were used as the excitation sources, each modulated by a mechanical chopper to generate the desired excitation square wave. The choppers allowed us to vary the modulation frequencies. The combined excitation wave was monitored by a photodiode (PD, PDA36A2, Thorlabs), while the generated upconversion emissions were detected by an avalanche photodiode (APD, APD410A2/M, Thorlabs). Further, for the PP-LID imaging demonstrations (Fig. 4), wide-field imaging was applied and a low-speed sCMOS camera (Hamamatsu ORCA-Flash4.0 v2) was instead used for detection. For the surface imaging experiments (Fig. 4), the image sequence was recorded with a short-pass filter with a cut-off at 950 nm (FF01-950/SP-25, OD6, Semrock). For the tissue imaging (Fig. 5 and Fig. S17), a laser-cleanup filter (FELH0950, Thorlabs, longpass filter, cut-on wavelength: 950 nm) was used on the excitation side, and image sequences were recorded with two short-pass filters with a cut-off at 950 nm (FF01-950/SP-25, OD6, Semrock) in combination with a band-pass filter at 810 nm (ET810/90, OD6, Chroma) on the detection side.

**Figure S6 Beat frequency (BF) signal generation of NaYF_4_:20%Yb^3+^, 2%Er^3+^@NaYF_4_ (YbEr@Y) UCNPs under dual square-wave excitation.** **(a)** The recorded excitation light signal from two merged 980 nm laser beams modulated at 51 and 53 Hz, respectively (excitation intensity: 30.5 W cm^-2^, each), and **(b)** its FFT magnitude spectrum. **(c)** The recorded generated upconversion emission signal (500-700 nm) of **YbEr@Y** UCNPs, and **(d)** its FFT magnitude spectrum. The dependence of **(e)** the defined BF weight (BFW) (eq. 2) and **(f)** the sum-frequency signal weight of the upconversion emission signal (500-700 nm) of **YbEr@Y** UCNPs on the average modulation frequency (${(f_{1}+f_{2})}/2$).

**Figure S7** Excitation-intensity dependence of the BFW of the UCL under dual sinusoidal-wave excitation obtained using the simple memoryless nonlinearity model detailed in section S2. The exponent $n$, representing the effective nonlinearity, was extracted from the excitation power dependence of the steady-state UCL shown in Fig. 1k in the main text.

**Figure S8** The dependence of the BFW of the UCL signal (430-850 nm) of **YbTm@Yb@Y** UCNPs on the average modulation frequency (${(f_{1}+f_{2})}/2$). Time averaged excitation intensity: 6 W cm^-2^, each laser.

**Figure S9 Optimization of the BF signal of UCNPs through nanoparticle engineering. (a)** Model describing upconversion emission pathways of NaYF_4_:20%Yb^3+^, 2%Er^3+^@NaYF_4_:20%Yb^3+^@NaYF_4_:30%Nd^3+^, 20%Yb^3+^ (**YbEr@Yb@YbNd**) core-multishell UCNPs under 980 or 808 nm excitation. **(b)** Dependence of the BFW of the upconversion emission signal (500-700 nm) of **YbEr@Yb@YbNd** UCNPs on the average modulation frequency (${(f_{1}+f_{2})}/2$) under dual square-wave excitation of two 980 nm laser beams (50% duty cycle, $f_{1}-f_{2}$ kept at 2 Hz, excitation intensity within pulses: 30.5 W cm^-2^, each). **(c)** Comparison of the BFWs of the **YbEr@Y**, **YbEr@Yb**, and **YbEr@Yb@YbNd** UCNPs under dual square-wave excitation of two 980 nm laser beams (excitation intensity within pulses: 30.5 W cm^-2^, each). **(d)** Dependence of the BFW of the upconversion emission signal (500-700 nm) of the same UCNPs and under the same conditions as in **(b)**, but now with one of the laser beams emitting at 980 nm replaced by a laser beam emitting at 808 nm.

**Figure S10 Excitation intensity dependence of YbTm@Yb@Y UCNPs** for the spectral emission range 750-850 nm

**Figure S11 Excitation intensity dependence of YbTm@Y UCNPs** for the spectral emission range 750-850 nm

**Figure S12 Excitation intensity dependence of YbEr@Yb@YbNd UCNPs** for the spectral emission range 500-700 nm

**Figure S13 Excitation intensity dependence of YbEr@Y UCNPs** for the spectral emission range 500-700 nm

**Figure S14 Excitation intensity dependence of YbEr@Yb UCNPs** for the spectral emission range 500-700 nm

**Figure S15** The bright-field image of the screw cap capped by NaYF_4_:20%Yb^3+^, 2%Er^3+^@NaYF_4_:20%Yb^3+^@NaYF_4_:30%Nd^3+^, 20%Yb^3+^ (**YbEr@Yb@YbNd**) UCNPs.

**Figure S16** The 23 Hz-filtered image of the screw cap capped by NaYF_4_:20%Yb^3+^, 2%Er^3+^@NaYF_4_:20%Yb^3+^@NaYF_4_:30%Nd^3+^, 20%Yb^3+^ (**YbEr@Yb@YbNd**) UCNPs.

**Figure S17 Photo of chicken tissue used in the experiments.** The red dashed line indicates the position of the UCNP-filled capillary.

**Figure S18** Spectrum of the laser radiation after transmission through a 950-nm long-pass laser-cleanup filter (FELH0950, Thorlabs). The inset shows a magnified view of the highlighted spectral region.

**Figure S19** Typical spectrum of the light measured with the laser-cleanup filter (FELH0950, Thorlabs) and the emission filter set-comprising two 950-nm short-pass filters (FF01-950/SP-25, OD6, Semrock) in combination with an 810-nm band-pass filter (ET810/90, OD6, Chroma)-in place, with UCNPs present and the 980-nm excitation lasers turned on. A UCNP-containing capillary (10 mg mL^-1^) was embedded in chicken tissue at a depth of approximately 5 mm. The excitation intensity was ~72 mW cm^-2^. The spectrum shown represents the average of 200 individual spectra acquired using an Ocean Optics QEPro spectrometer with an exposure time of 2000 ms.

**Figure S20** Typical spectrum of the light measured with the laser-cleanup filter (FELH0950, Thorlabs) and the emission filter set—comprising two 950-nm short-pass filters (FF01-950/SP-25, OD6, Semrock) in combination with an 810-nm band-pass filter (ET810/90, OD6, Chroma)—in place, recorded without UCNP inclusion while the 980-nm excitation lasers remained on. The excitation intensity was ~72 mW cm^-2^. The spectrum shown represents the average of 200 individual spectra acquired using an Ocean Optics QEPro spectrometer with an exposure time of 2000 ms.

**Figure S21 (a)** A typical frame in the image sequence (video 2) of a capillary filled with **YbTm@Yb@Y** (10 mg mL^-1^) embedded in chicken breast tissue at an approximate depth of 5 mm under dual square-wave excitation of two 980nm laser beams, measured with varying ambient light levels. Time-averaged excitation intensity: 35 mW cm^-2^, for each laser beam. Frame exposure time: 400 ms. A laser-cleanup filter (FELH0950, Thorlabs, longpass filter, cut-on wavelength: 950 nm) was used on the excitation side. Image sequence was recorded with two short-pass filters with a cut-off at 950 nm (FF01-950/SP-25, OD6, Semrock) in combination with a band-pass filter at 810 nm (ET810/90, OD6, Chroma). **(b)** Time trace of integrated intensity values from the image sequence in video 2. **(c)** The FFT magnitude spectrum of the time trace of integrated signals in **(b)**. **(d)** The averaged image of the sample (UCNP-filled capillary behind chicken tissue), obtained from the recorded image sequence in video 2. **(e)** The BF (1 Hz)-filtered image. **(f)** Emission profiles along the marked lines in the averaged (**d**) and 1 Hz-filtered (**e**) images. Profiles in **(f)** are normalized to their own maximum values.

**Figure S22 (a)** A typical frame in the image sequence of a capillary filled with **YbTm@Yb@Y** (100 µg mL^-1^) embedded in chicken breast tissue at an approximate depth of 2 mm under dual square-wave excitation of two 980nm laser beams. Time-averaged excitation intensity: 255 mW cm^-2^, for each laser beam. Frame exposure time: 4 s. A laser-cleanup filter (FELH0950, Thorlabs, longpass filter, cut-on wavelength: 950 nm) was used on the excitation side. Image sequence was recorded with two short-pass filters with a cut-off at 950 nm (FF01-950/SP-25, OD6, Semrock) in combination with a band-pass filter at 810 nm (ET810/90, OD6, Chroma). **(b)** Time trace of integrated intensity values from the image sequence. **(c)** The FFT spectrum of the time trace of integrated signals in **(b)**. **(d)** The averaged image of the sample (UCNP-filled capillary behind chicken tissue), obtained from the recorded image sequence. **(e)** The BF (0.1 Hz)-filtered image. **(f)** Emission profiles along the marked lines in the averaged (**d**) and 0.1 Hz-filtered images. Profiles in **(f)** are normalized to their own maximum values.

**Figure S23** Excitation-intensity dependence of the 800 nm upconversion emission band of YbTm@Yb@Y UCNPs. UCNP inclusion embedded in chicken breast tissue at a depth of 3 mm under continuous-wave 980 nm excitation. The excitation intensities were the values on the tissue surface.
